# A Parallel Cross Convolutional Recurrent Neural Network for Automatic Imbalanced ECG Arrhythmia Detection with Continuous Wavelet Transform

**DOI:** 10.3390/s22197396

**Published:** 2022-09-28

**Authors:** Tabassum Islam Toma, Sunwoong Choi

**Affiliations:** School of Electrical Engineering, Kookmin University, Seoul 02707, Korea

**Keywords:** cardiac arrhythmia, convolutional neural network, continuous wavelet transform, electrocardiogram, recurrent neural network

## Abstract

Automatic detection of arrhythmia using electrocardiogram (ECG) and deep learning (DL) is very important to reduce the global death rate from cardiovascular diseases (CVD). Previous studies on automatic arrhythmia detection relied largely on various ECG features and have achieved considerable classification accuracy using DL-based models. However, most previous research has ignored multi-class imbalanced problems in ECG arrhythmia detection. Therefore, it remains a challenge to improve the classification performance of the DL-based models. This paper proposes a novel parallel cross convolutional recurrent neural network in order to improve the arrhythmia detection performance of imbalanced ECG signals. The proposed model incorporates a recurrent neural network and a two-dimensional (2D) convolutional neural network (CNN) and can effectively learn temporal characteristics and rich spatial information of raw ECG signals. Continuous wavelet transform (CWT) is used to transform the ECG signals into a 2D scalogram composed of time–frequency components, and subsequently, the 2D-CNN can learn spatial information from the 2D scalogram. The proposed model is not only efficient in learning features with imbalanced samples but can also significantly improve model convergence with higher accuracy. The overall performance of our proposed model is evaluated based on the MIT-BIH arrhythmia dataset. Detailed analysis of evaluation metrics reveals that the proposed model is very effective in arrhythmia detection and significantly better than the existing hierarchical network models.

## 1. Introduction

Cardiovascular diseases (CVD) have become a global concern because of their deadly consequences, either death or disability which severely affects people’s life. A report published by the World Health Organization (WHO) manifests that approximately 17.9 million people died from CVD in 2019, accounting for 30% of global deaths [1]. Recently, WHO has warned mass people announcing CVD as the top ten health threats in the world and it is estimated that the death toll caused by CVD will be increased to 23 million by 2030. Cardiac arrhythmia, which causes heart rhythmic problems, is the fundamental disease group of CVD. Due to coronary artery disease, high blood pressure, changes in heart muscle, and valve disorders, electrical pulses that coordinate heartbeats can cause the heart to beat irregularly, resulting in cardiac arrhythmia [2]. According to reports, some forms of arrhythmia, such as ventricular fibrillation and tachycardia, are outrageous and life-threatening and can trigger cardiac arrest and sudden death. Therefore, beat-by-beat examination utilizing electrocardiogram (ECG) has become a vital modern medical tool for early diagnosis of cardiovascular conditions, such as the process of cardiac excitability, transmission, and recovery.

The ECG is used to record the electrical activities and states of the heart over time through electrodes attached to the skin surface [3]. Thus, the presence of abnormal heart electrical activity can be detected by analyzing the ECG, which can assist to derive the type of arrhythmia in turn. Previously, the ECG analysis as well as arrhythmia recognition and the diagnosis was performed with the help of human intervention. However, this procedure is very laborious and time-consuming [4]. Recently, the emergence of intelligent healthcare requires the incorporation of artificial intelligence to automatically detect irregular heart rhythms from the ECG. Over the past decade, many researchers have investigated and developed several schemes for automatic arrhythmia detection [5,6,7,8,9,10]. Although these developed schemes could achieve higher accuracy on the ECG signal classification, researchers also investigated their performances by introducing minor variations in the machine learning (ML) classifier and fusion of different hand-crafted features in [11,12,13]. A disease specific feature-based ECG heartbeat classification procedure was developed in [11]. A one-versus-one rule based binary support vector machine (SVM) classifier was used to perform classification. In [12], a novel ECG heartbeat classification method was proposed that combines morphological features and RR interval information. An SVM classifier was used to perform the classification in this paper. A combination of projected and dynamic features was used to classify the ECG heartbeat in [13]. A random projection matrix was employed to extract projected features, while three weighted RR intervals were considered as the dynamic features. Finally, using those two kinds of features, an SVM classifier was used to cluster heartbeats into one of fifteen or five classes. However, these schemes are based on ML algorithms (e.g., neural network, SVM, etc.), with various features, including hand-crafted time and frequency features extracted empirically, which require extensive professional knowledge. In addition, it becomes more difficult to select the model parameter when the feature dimension increases.

With the development of the deep learning (DL) algorithm, it has become predominant in the ECG classification because of its exemplary performance, especially that of convolutional neural network (CNN) using one-dimensional (1D) and two-dimensional (2D) convolution. The ability of DL-based models to automatically learn invariant and hierarchical features directly from data has increased their applicability in designing end-to-end learning mechanisms that take data as input and class prediction as output. Recent DL-based models have been developed depending on the 1D ECG signal or 2D representation of ECG by transforming the ECG signal into images or some matrix form. Numerous studies on 1D ECG classification have recently been conducted [14,15,16,17,18,19,20]. The authors demonstrated a performance comparison among five models: 1D-CNN, CNN combined with linear discriminant analysis, SVM classifier, CNN combined with SVM, and CNN combined with SVM and RR interval input in [14]. A 1D-CNN classifier was designed to identify atrial fibrillation in [15]. Before using the ECG recordings as input, Nurmaini et al. [15] used the discrete wavelet transform to remove noise from the ECG recordings. In [16], Acharya et al. developed a typical nine-layer 1D-CNN model to classify five categories of heartbeats, i.e., non-ectopic, supraventricular ectopic, ventricular ectopic, fusion, and unknown beats. The authors performed filtering, R-peak extraction, and heartbeat segmentation before feeding the data into the 1D-CNN classifier to identify heartbeat categories. In another study, an 11-layer 1D-CNN model was proposed to detect five types of arrhythmias, and its overall performance was investigated by Acharya et al. [17]. At this time, their experiment included ECG recording durations of 2 and 5 s instead of heartbeats. However, adopting a hybrid model and variations of CNN can further improve the performance of the classic CNN. The authors of [18] proposed a neural network-based method that involves two steps for precise classification of heartbeats by following the Association for the Advancement of Medical Instrumentation (AAMI) inter-patient standards. Data preprocessing and features extraction were the task of the first step, while the classification was performed by a two-layer classifier in which each layer consists of two independent fully connected neural networks in the second step. The experimental results depicted that the proposed model precisely detects arrhythmia conditions. In [19], Chen et al. proposed a DL-based hybrid model that combines CNN and long short-term memory (LSTM) network to classify heartbeats. Their model requires the input of two forms of 1D data from the ECG signals, namely the ECG heartbeats that provide the morphological information and the adjacent segment of the ECG beats that enrich temporal information. In another study [20], a complex DL-based model consisting of a CNN and an LSTM network was developed to detect six types of ECG signals by processing 10 s ECG slices of the MIT-BIH arrhythmia dataset. Experimental results validated that the proposed model was very effective in automatically detecting arrhythmia. However, the outstanding performance of 2D-CNN on image classification or pattern recognition attracted researchers to convert raw ECG data to images and utilize them for precise arrhythmia detection. Fourier transform is a well-known and widely used method for extracting time–frequency components from nonstationary ECG signals. [21] studied the performance of time–frequency spectrogram-based 2D-CNN model on ECG classification. In this study, the authors utilized the short-time fourier transform method to convert ECG signals into time–frequency spectrograms. An elaborate exhibition of the experiment proves that 2D-CNN can achieve higher classification accuracy than 1D-CNN. In [22], spectro-temporal images were used to train and optimize a dense CNN. The developed model in [22] can achieve significantly improved performance by capturing both beat-to-beat and single-beat information. Another widely accepted technique to generate time–frequency representation from a given segment of the ECG is wavelet transform (WT). The authors in [23] adopted WT to transform heartbeat time intervals of ECG signals into images and then trained a six-layer CNN employing these images for heartbeat classification. Very recently, a generative adversarial neural network has become popular in the field of image super-resolution and image reconstruction. The capability of image reconstruction to convert the raw 1D ECG signal data into a 2D image has been exploited in [24]. Afterward, the authors fed these images as input to DenseNet which produces highly accurate classification with high sensitivity and specificity using four classes. In [25], treating multi-lead ECG as 2D matrices for input, a novel model called multi-lead-CNN (ML-CNN) was designed, which employs sub-2D convolutional layers and lead asymmetric pooling layers. Incorporating both hand-crafted RR interval and spatial features extracted by CNN from the 2D scalogram, a novel method was developed in [26] for AAMI standard-based ECG heartbeat classification. In this study, their method shows poor performance for the highly skewed class.

Despite the large number of studies in the area of arrhythmia detection exploiting the ECG signal and DL-based models, there are still many challenges because of the differences in the recording environment, variations of disease patterns among the subjects during testing, complex, nonstationary, and noisy nature of the ECG signal. Moreover, the aforementioned methods ignored multi-class imbalanced problems in arrhythmia detection. Imbalanced data learning [27] has always been a concerning issue in the field of ML, and the adverse effect of data imbalance on the performance of the learning algorithms is not negligible. Data imbalance issues also exist in the field of arrhythmia detection from the ECG data analysis [28]. Therefore, solving the imbalance problem can improve the overall performance. Considering the data imbalance problem, [28] proposed a novel multi-module neural network system AAMI standard-based ECG heartbeat classification. To tackle the imbalance problem, the authors introduced the borderline-SMOTE algorithm, a novel context feature module, and two-phase training (referred to as 2PT). In [29], a transfer learning-based heartbeat classification method utilizing 2D time–frequency diagrams was proposed. The authors employed a hybrid time–frequency analysis of the Hilbert transform and the Wigner–Ville distribution to convert 1D ECG recordings into 2D time–frequency diagrams.

However, it can be noticed from the aforementioned studies ([19,20,22,26,28]) that multi-type feature fusion, while training the DL-based model, can lead to improved performance. In a recent work [30], the authors developed a parallel cross CNN to improve the detection performance of imbalanced traffic flows by fusing the flow features learned from two-branch CNNs. Motivated by this fact, this paper proposes a novel parallel cross-convolutional recurrent neural network to improve the arrhythmia detection performance of imbalanced ECG signals. The proposed model consists of two branches: a recurrent neural network (RNN) and a 2D CNN for temporal characteristics and spatial features. Three LSTM layers and three convolutional blocks are used to form the top and bottom branches of the model, respectively. After each block and layer operation, the features are fused and passed through the subsequent layers and blocks. Hence, the model becomes more distinguishable and robust for imbalanced ECG signal classification, especially for the AAMI standard-based classes, which are largely skewed. The main contributions of this paper are summarized as follows:

▪ Considering ECG arrhythmia detection as a multi-class imbalance problem, this paper proposes a novel parallel cross convolutional RNN classifier to improve detection performance.
▪ The proposed model includes two branches consisting of the LSTM network and CNN to capture both temporal characteristics from the ECG segment and spatial characteristics from the 2D scalogram. The network becomes more distinguishable and robust through cross-fusion of the features.
▪ Continuous wavelet transform (CWT) has been adopted to convert the preprocessed ECG segments into 2D scalograms. Subsequently, both ECG segment and corresponding 2D scalogram have been used as input for training and testing the proposed model.
▪ Finally, we have evaluated the performances of the proposed model on the MIT-BIH arrhythmia dataset. We also compared the performance with other existing works and investigated the performance by varying the wavelet function, such as Gaussian wavelets of order 8 (*gaus8*) and order 4 (*gaus4*), Mexican hat wavelet (*mexh*), Morlet wavelet (*morl*).

The remainder of this paper is structured as follows: Section 2 describes the overall methodology of ECG arrhythmia detection along with the details of data preprocessing and the proposed model used in this paper. In Section 3, the experimental results followed by implementation details have been elaborately illustrated. Finally, we draw the conclusions of this paper in Section 4.

## 2. Methodology

**Figure 1:**
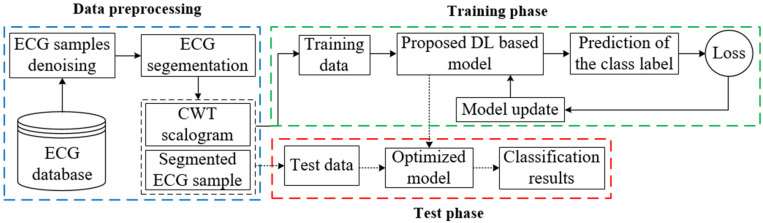
The architecture of arrhythmia recognition and classification.

This section describes ECG arrhythmia detection exploiting a deep neural network if a set of ECG samples and their corresponding arrhythmia classes are given. Figure 1 depicts the architecture of the proposed model for ECG arrhythmia classification method. This method includes several steps before enabling the proposed model to use ECG samples for training. Each essential component of arrhythmia detection is elaborately demonstrated in the following sections.

### 2.1. ECG Signal Preprocessing and Segmentation

**Figure 2:**
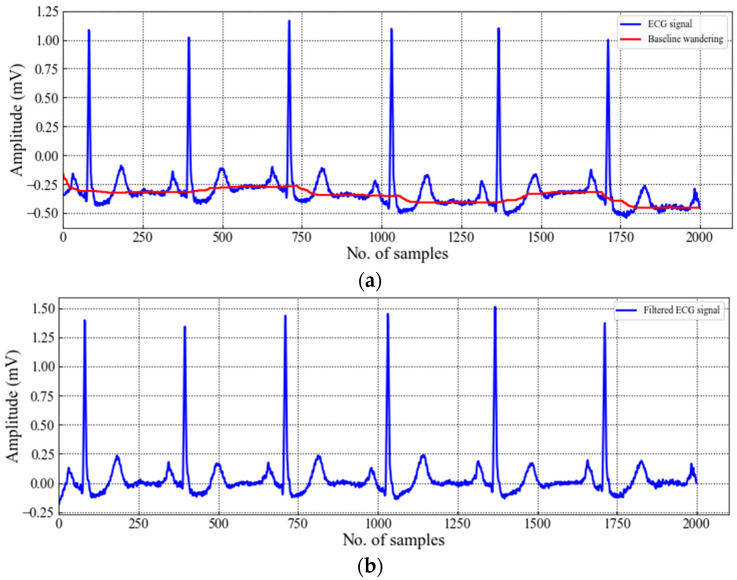
ECG signal denoising (**a**) before filtering and (**b**) after filtering.

When raw ECG data are collected in the clinical environment, the data contains various types of noise, including baseline wandering, electromyogram signals, and power-line disturbance [26]. Without removing the noise contamination, the extracted features may lead to performance degradation. Therefore, filtering is required to denoise the ECG signal before segmentation. However, attention should be provided when filtering is applied, otherwise the ECG signal may lose useful information. In this study, the baseline wandering technique has been adopted for noise removal. To obtain the baseline, two consecutive filters, including a 200 ms width median filter followed by a 600 ms median filter, are utilized. Next, the computed baseline is subtracted from the raw ECG signal. Various studies show that the median filter is effective in outlier elimination without distorting the phase of the signal compared with other filter techniques, such as regular infinite impulse response and finite impulse response. Figure 2 depicts the ECG signal filtering step.

**Figure 3:**
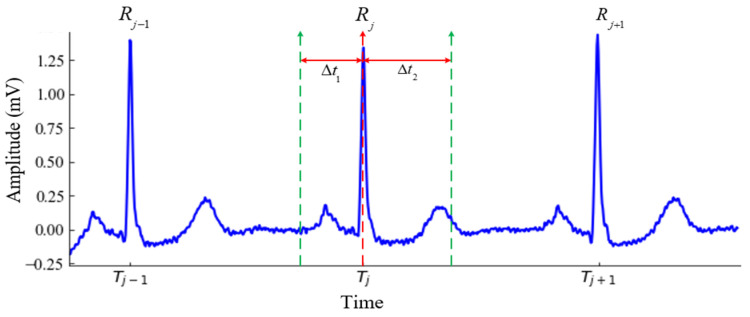
ECG heartbeat segmentation.

The ECG signal segmentation involves the extraction of a complete heartbeat from the ECG signal. This step usually requires the detection of position of R-peak because R-peak is regarded as an anchor point for locating a complete heartbeat [31]. In this study, no algorithm is adopted for R-peak detection rather we have exploited the annotated R-peak location to segment the ECG signal into a series of heartbeats. Figure 3 depicts an example of a heartbeat segmentation from the ECG signal. The preceding and succeeding time lengths, $\Delta t_{1}$ and $\Delta t_{2}$, respectively, of any R-peak $R_{j}$ help determine the length of an ECG segment. Consequently, the number of data samples involved in each segment can be calculated as follows:

(1) $$N_{sample}=\left(\Delta t_{1}+\Delta t_{2}\right)\times f+1\;$$

For each heartbeat segment, we have obtained $\Delta t_{1}=$ 0.3 s and $\Delta t_{2}=$ 0.5 s in this study.

### 2.2. Scalogram Obtained Using CWT

The ECG signal is nonstationary data composed of time-varying frequency components. Therefore, time–frequency representation of an ECG segment can be a useful tool for arrhythmia detection. STFT is one of the most popular and widely used techniques for transforming the ECG signal into a 2D time–frequency image. The STFT uses a fixed-size window function when it converts the ECG signal into time–frequency image. However, choosing the size of the window function often limits its effectiveness in many areas [32]. To overcome this limitation, CWT has been explored in different application areas in recent years. Unlike the STFT method, CWT can choose a smooth analytical mother wavelet that can identify the dynamic frequency properties of ECG signals at different scales.

In addition, CWT can provide high-time resolution and low-frequency resolution in high frequencies, as well as high-frequency resolution and low-time resolution in low frequencies, by adjusting the scale and translation parameters [33]. Given an ECG signal x(t), the CWT is defined as the inner product between the signal and the mother wavelet ψ(t) that yields the 2D matrix of wavelet coefficients $C_{a,b}$ as follows:

(2) $$C_{a,b}=\frac{1}{\sqrt{a}}\int_{-\infty }^{\infty }x\left(t\right)\psi \left(\frac{t-b}{a}\right)dt\;$$

where a represents the scale parameter, b is a time-shifting or translation parameter, and ψ(t) is the mother wavelet function. The scale can be replaced by frequency as follows:

(3) $$F=\frac{f_{c}\times f_{s}}{a}$$

where $f_{c}$ and $f_{s}$ represent the center frequency of the mother wavelet and the sampling frequency of the signal x(t), respectively. However, choosing the mother wavelet function is crucial because, depending on the application areas, the classifier’s performance largely relies on the mother wavelet function. In this paper, four wavelet functions, namely *gaus8*, *gaus4*, *mexh*, and *morl,* have been employed to generate a 2D scalogram and to distinctly investigate the performance of the proposed model. The mathematical expression for *gaus8*, *gaus4*, *mexh*, and *morl* wavelet functions can be represented as follows [34,35,36]:

(4) $$\psi_{gaus}\left(mt\right)=C_{m}\frac{d^{m}}{dt^{m}}\;g_{c}\left(t\right)$$

**Figure 4:**
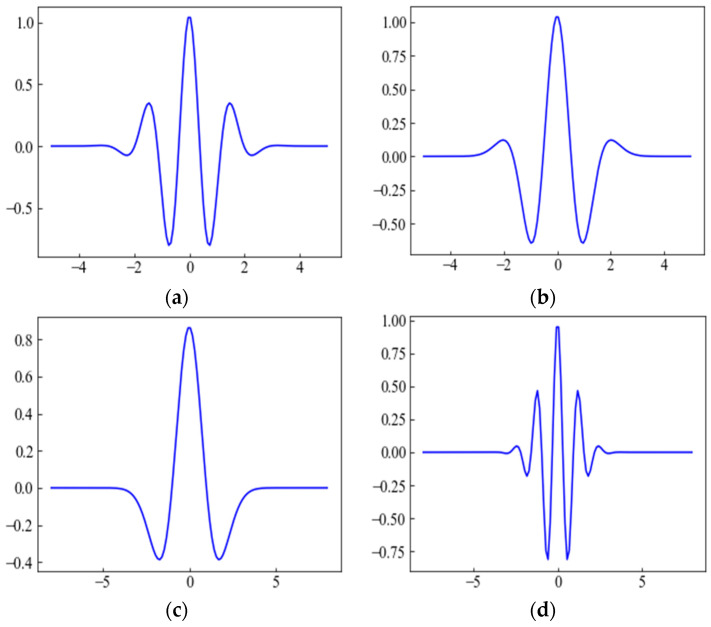
Continuous wavelet functions. (**a**) Gaussian of order 8 (*gaus8*), (**b**) Gaussian of order 4 (*gaus4*), (**c**) Mexican hat (*mexh*), and (**d**) Morlet (*morl*).

(5) ψmexh(t)=e2πite−t22σ2

(6) ψmorl(t)=(1−t2σ2)e−t22σ2

where $\psi_{gaus}\left(mt\right),$ $\psi_{mexh}\left(t\right),\;$and $\;\psi_{morl}\left(t\right)$ denote Gaussian wavelet, *mexh*, and *morl*, respectively. The parameter m denotes the order of the Gaussian wavelet and $g_{c}\left(t\right)$ denotes Gabor function, which is the product of the Gaussian function and the complex exponential function. Figure 4 shows the four types of wavelet functions.

### 2.3. Model Description

**Figure 5:**
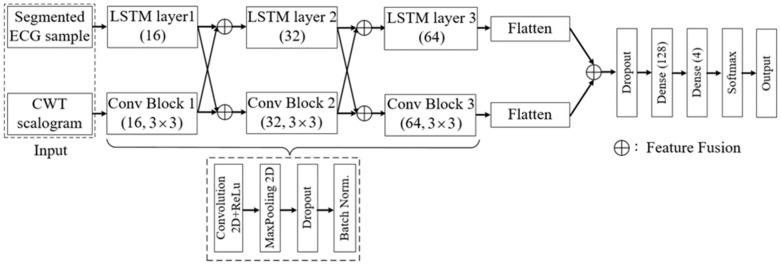
Architecture of the proposed parallel cross convolutional recurrent neural network.

**Figure 6:**
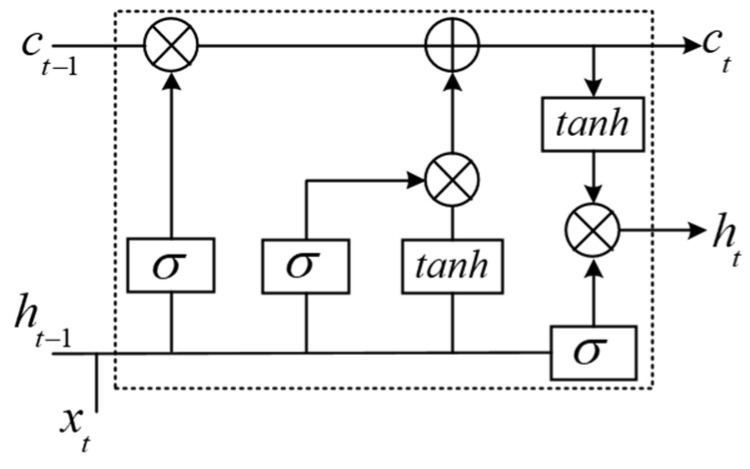
Architecture of the LSTM cell internal structure.

The proposed model is composed of two branches, as shown in Figure 5. The top branch comprises several RNN layers to detect temporal changes within a given heartbeat. The RNN layer exploits cell mechanisms to remember the previous pattern of a heartbeat signal and utilize it to capture the next-step temporal characteristics. More specifically, we have used the LSTM layer to construct the top branch. LSTM is a variant of RNN, which includes memory cells to overcome the vanishing gradient of the RNN and is particularly suitable for processing time series signals [37]. There are three LSTM layers in the top branch; each contains 16, 32, and 64 output units, respectively. Each LSTM layer is composed of several LSTM cells. Figure 6 depicts the internal structure of the LSTM cell. For an ECG signal x(t) at time *t*, the corresponding output h(t) and internal state c(t) can be calculated as follows:

(7) $$f\left(t\right)=\sigma \left(\omega_{f}\cdot \left[h\left(t-1\right),x\left(t\right)\right]+\beta_{f}\right)$$

(8) $$i\left(t\right)=\sigma \left(\omega_{i}\cdot \left[h\left(t-1\right),x\left(t\right)\right]+\beta_{i}\right)$$

(9) $$o\left(t\right)=\sigma \left(\omega_{o}\cdot \left[h\left(t-1\right),x\left(t\right)\right]+\beta_{o}\right)$$

(10) $$c\left(t\right)=f\left(t\right)⊙c\left(t-1\right)+i\left(t\right)⊙tanh\left(\omega_{c}\cdot \left[h\left(t-1\right),x\left(t\right)\right]+\beta_{c}\right)$$

(11) h(t)=o(t)⊙tanh(c(t))

where f(t), i(t), and o(t) denote the forget, input, and output gates of the LSTM cell, respectively, which are jointly utilized to control the update of the h(t). More specifically, f(t) assists in removing useless information contained in LSTM’s past state, i(t) controls the addition of information to LSTM’s current state, and o(t) determines the final output. Due to these three gates, the temporal characteristics in the ECG signal can be effectively extracted. 

The bottom branch is composed of several convolutional blocks. Each block comprises a 2D convolutional layer with a rectified linear unit (ReLu) activation function, a 2D max-pooling layer, a dropout layer, and a batch normalization layer. All of the convolutional layers use (3×3) 2D convolutional filter to extract spatial features. The number of convolutional filters at each layer is 16, 32, and 64, respectively. A (2×2) max-pooling layer is deployed after each convolutional layer to extract more representative features, reducing the size of feature maps. In addition, to alleviate the bottom branch from overfitting during training, a dropout layer with a dropout rate of 0.1 is inserted in every convolutional block. The last layer of each convolutional block is the batch normalization layer. Batch normalization is considered another type of regularization technique that enables a network to converge faster, avoiding overfitting. The overall configuration of our proposed model is shown in Table 1.

The proposed model combines high-level spatial features extracted from the CWT scalogram with the temporal characteristics of the upper layers to improve arrhythmia detection results. The model performs three feature fusion operations on the feature maps. The number of output units in the LSTM layers and the number of filters in the convolutional blocks (e.g., 16, 32, 64) are taken in such a way that the pairs of the feature map after each LSTM layer and convolutional block can be concatenated by applying the feature fusion technique. For the first two pairs of the feature map, a cross-fusion operation is adopted so that the fused features can be passed through both convolutional blocks and LSTM layers. The first two fusion operations do not change the size of the feature map, only doubling the number of channels. The last pair of the feature map is flattened before performing a linear fusion operation. The final fused feature is then passed through a dropout layer (with a dropout rate of 0.1) and a dense layer to reduce the size of the fused feature. The network’s output uses the dense and softmax layers to perform multi-class imbalanced ECG arrhythmia classification.

## 3. Experimental Results

This section first describes the ECG dataset and evaluation metrics for evaluating the proposed model performances. Finally, the overall performances, along with the comparison with other existing network performances, have been presented to investigate the effectiveness of the proposed model and unveil its superiority over other existing networks.

### 3.1. ECG Dataset Description

**Figure 7:**
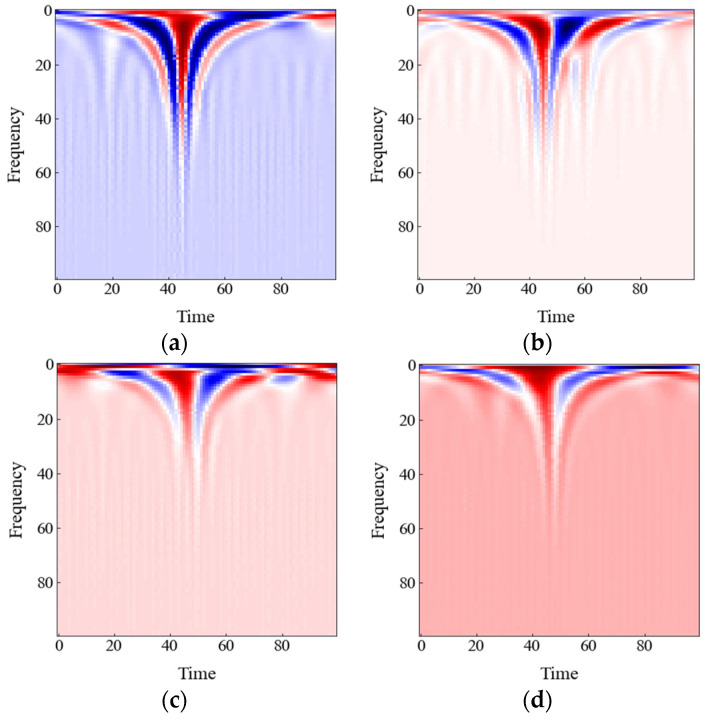
Scalogram of four types of ECG classes based on the AAMI standard (**a**) NB, (**b**) SVEB, (**c**) VEB, and (**d**) FB.

Although many open-source datasets are available for research in the field of arrhythmia detection, the ECG signal used in this paper is taken from the well-known MIT-BIH arrhythmia database [38]. The database contains 48 fully annotated half-hour two-lead ECG recordings sampled at 360 Hz obtained from 47 subjects. Twenty-three recordings are randomly selected from the subjects’ daily routine. The remaining 25 recordings, containing less common but clinically significant arrhythmias, are also obtained from the above stated ambulatory ECG recordings. Each recording involves two channels of ECG data with 11-bit resolution over a 10-mV range signal. The first channel includes modified lead II (MLII), while the other channel includes lead depending on the recording (e.g., VI, VII, V2, V4, or V5). In the experiment, ECG signals obtained from the MLII channel are used because MLII is ubiquitous in the above records. Two or more cardiologists carefully investigated these recordings and labeled them into 15 arrhythmia types (e.g., normal beat, left bundle branch block beat, right bundle branch block beat, atrial escape beat, nodal (junctional) escape beat, atrial premature beat, aberrant atrial premature, nodal (junctional) premature, premature or supraventricular premature, premature ventricular contraction, ventricular escape, fusion of ventricular and normal, fusion of paced and normal, paced beat, and unclassifiable beat). Recommended by the AAMI standards, all 15 types of ECG heartbeat labels are mapped to 5 AAMI categories, i.e., non-ectopic cardiac beat (NB), supraventricular ectopic beat (SVEB), ventricular ectopic beat (VEB), fusion beat (FB), and unknown beat (QB), as shown in Table 2. However, four ECG recordings (i.e., 102, 104, 107, and 217), which include paced beats, as well as the QB class, are discarded in this paper, similar to other studies [26,39,40]. As we described above, CWT is applied to transform the segmented ECG heartbeat into a 2D scalogram, Figure 7 illustrates the scalogram, decomposed by *gaus8* wavelet function, of four types of ECG heartbeat. A total of 100 continuous values ranging from 2.16 to 216 are used as the scaling factor of the mother wavelet. Each of the scalograms in this study is resized into a (100 × 100) matrix in order to feed into our proposed classifier. The scalograms are represented as a function of time and frequency in the figure. 

The spike in the scalograms indicates that there is a change in the frequency of the ECG signal. It is also evident from the depth of the spike that the change in frequency is not similar for all four classes. Therefore, the distinct pattern of these scalograms can be very impactful, and carrying out the classification with these scalograms can lead to satisfactory performances.

### 3.2. Implementation Details and Performance Metrics

After having the preprocessed ECG heartbeats along with their corresponding 2D scalograms, 80% of each category are selected for the training set, and the remaining 20% are used for the test set. After splitting, the number of total training samples is 80,401, and that of total testing samples is 20,100, including all four AAMI categories. Next, the classifier is trained and optimized using the training data samples and an optimizer. Finally, the performance has been evaluated using the test data samples. All training and testing programs have been performed in an Anaconda Python 3.7 environment on a system equipped with a 3.80 GHz CPU, 256 GB RAM, and a single Nvidia Quadro RTX 6000 GPU. All hyper-parameters used to train the proposed model are summarized in Table 3. However, varying the learning rate (*lr*), the performance of the proposed model has been evaluated at the beginning. Keeping the batch size = 512, the performances for three *lr* (e.g., 0.0005, 0.0001, and 0.00005) are investigated. *Adam* optimizer, along with categorical-cross entropy loss function, is used during the training of the proposed model. Furthermore, 60 iterations or epochs are used to optimize the network. 

Afterward, the optimum model performance has been compared with those of six existing studies. Finally, the classifier performance is also demonstrated by changing the wavelet functions (e.g., *gaus8*, *gaus4*, *mexh*, and *morl*).

To evaluate the performances, four standard statistical indices, also known as evaluation metrics (e.g., accuracy (ACC), positive predictive value (PPV), sensitivity (SE), and F1-score (F1)), are used in this study. These metrics are defined as follows:

(12) $$ACC_{i}=\frac{TP_{i}+TN_{i}}{TP_{i}+TN_{i}+FP_{i}+FN_{i}}$$

(13) $$PPV_{i}=\frac{TP_{i}}{TP_{i}+FP_{i}}$$

(14) $$SE_{i}=\frac{TP_{i}}{TP_{i}+FN_{i}}\;$$

(15) $$F1_{i}=\frac{2PPV_{i}\cdot SE_{i}}{PPV_{i}+SE_{i}}$$

where $TP_{i}$ and $FN_{i}$, also known as true positive and false negative, respectively, refer to the number of the i-th class correctly predicted and the number of the i-th class classified into other classes, respectively. In addition, $TN_{i}$ and $FP_{i}$, also known as true negative and false positive, respectively, are the number of other classes that are not classified as the i-th class and the number of other classes predicted as the i-th class, respectively.

### 3.3. Performance Analysis

**Figure 8:**
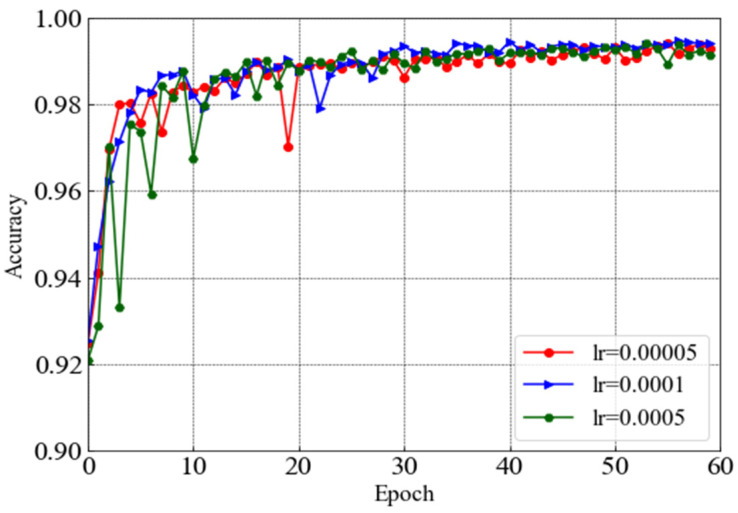
Accuracy value curves for different learning rates.

To investigate the convergence performance of the proposed model, we depicted the accuracy value curves for different *lr*, setting the batch size as 512 and wavelet as *gaus8* in Figure 8. It can be seen that *lr =* 0.0001 helps converge with higher accuracy compared with the other two *lr* (e.g., 0.0005 and 0.00005) after 60 iterations. For the other two values of *lr* (e.g., 0.0005 and 0.00005), multiple numbers of fluctuations are observed while *lr =* 0.0001 can attain a smaller number of fluctuations, indicating that the learning process for the proposed model is smooth and the convergence of the proposed model is better for the *lr =* 0.0001 value.

**Figure 9:**
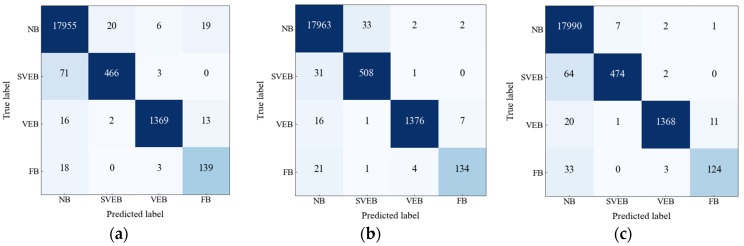
Confusion matrices for (**a**) *lr* = 0.0005, (**b**) *lr* = 0.0001, and (**c**) *lr* = 0.00005.

Figure 9 shows the confusion matrices for *lr* values. In Table 4, we compute the performance of the proposed model numerically in terms of four evaluation metrics taking the values of $TP_{i}$,$\;FP_{i}$, $TN_{i}$, and $FN_{i}$ from the confusion matrices. The last four rows of the table compute the average value for all metrics. The average value of the evaluation metric reveals that the proposed model achieves the highest average value for PPV, SE, and F1 scores for batch size = 512 and *lr* = 0.0001. Table 5 shows 10-fold cross validation score for the proposed classifier. In this process, the whole data are split into ten number of folds and one fold is considered for testing and the rest will be for training and moving on with iterations in the entire process. In this study, 10-fold cross validation is applied to justify the proposed model generalization ability. From the table, it can be seen that the difference between the average value of the performance metrics (e.g., PPV, SE, F1) for all the four classes after applying 10-fold cross validation and the actual value of the performance metrics is very small. 

This outcome proves that the proposed classifier is generalizing and actively learning.

A comparison of the performances with other existing works [11,12,14,26,28,29] has been provided in Table 6. Based on the average value, it can be stated that the proposed model achieves outstanding performance compared to those of studies mentioned in the table in terms of all evaluation metrics. In terms of performance metrics, the proposed model can attain an average improvement of 1.45–3.57%. However, it is noticeable that achieving higher performance for the FB class is difficult. Since the FB class is composed of the fusion of ventricular and normal beat, which is very close to the normal heartbeat, existing studies predict a large number of ECG samples from the FB class as NB class. Therefore, the overall performance in terms of ACC, PPV, SE, and F1 scores for the FB class becomes deteriorated. In our study, the proposed model allows fused temporal characteristics and rich spatial features to be passed through the LSTM layers and convolutional blocks; hence, the model becomes more robust and distinguishable for the FB class. From the confusion matrix, it can be seen that a few ECG samples from the FB class are misclassified as NB class, resulting in higher ACC, PPV, and SE. Thus, our model shows more promising results for the FB class than that of other works, as shown in Table 6. For this class, our model has achieved a 5.46% improvement in the F1 score than [29]. 

**Figure 10:**
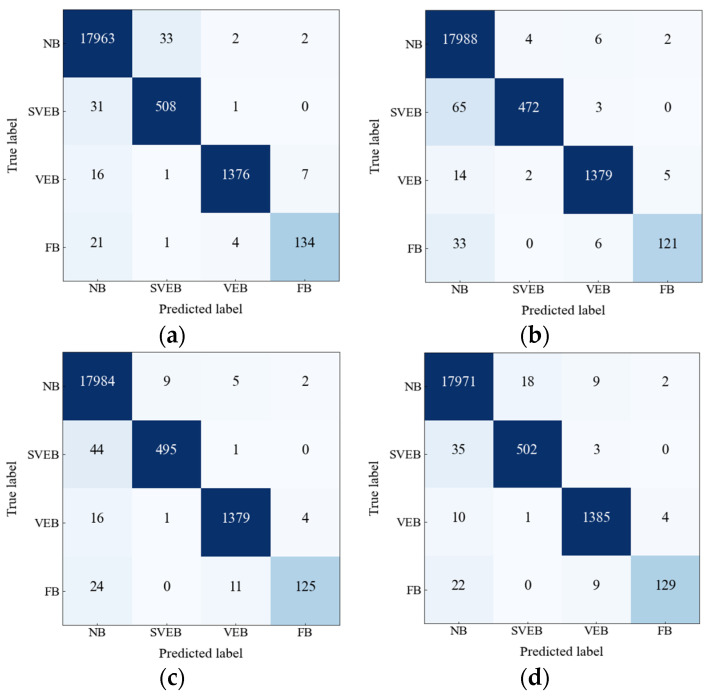
Confusion matrices for four wavelet functions (**a**) *gaus8*, (**b**) *gaus4*, (**c**) *mexh*, and (**d**) *morl* keeping *lr =* 0.0001.

In this paper, we have also observed and analyzed three other wavelet functions’ performances in the ECG classification. Taking the values of $TP_{i}$,$\;FP_{i}$, $TN_{i}$, and $FN_{i}$ from the confusion matrices depicted in Figure 10, a comparison of four wavelet functions’ performances is listed in Table 7. Significant variation among the performance of the four types of wavelet function for all metrics except that of ACC appeared. Specifically, *gaus8* performs better than other wavelets for the VEB and FB classes, while *mexh* performs better for the SVEB class. Since the number of samples is large in the NB class, the performance is almost the same for all the wavelets. Because of the higher similarity between the waveform of the ECG signal and the *gaus8* wavelet function, the *gaus8* based scalogram is the most suitable for arrhythmia classification. 

Therefore, this investigation recommends that using a wavelet function close to the ECG signal (e.g., *gaus8*) will lead to the best ECG arrhythmia detection performance.

## 4. Conclusions

In this article, we proposed a novel parallel cross convolutional RNN to improve the arrhythmia detection performance by modeling ECG signal classification as an imbalanced problem. The proposed model incorporates extracted temporal characteristics and spatial features, utilizes several LSTM layers in the top branch and several convolutional blocks in the bottom branch, and allows for flowing throughout the whole model, making the model’s learning more distinguishable and robust. We have evaluated the detailed performance of the proposed model in terms of four evaluation metrics on the well-known MIT-BIH dataset. Additionally, a comparison of the performances of other existing works with the optimum performance of the proposed model has been presented. The proposed model achieves higher performances compared with others, especially since the classifier shows an outstanding performance for the FB class. Finally, the model’s performance depending on the four distinct wavelet functions has been elaborately described. Our proposed classifier can distinguish NB, SVEB, VEB, and FB classes with an average ACC, average PPV, average SE, and average F1 of 99.58%, 96.60%, 93.97%, and 95.22%. For the most skewed class, i.e., FB class, we have obtained promising results (*PPV* = 93.71%, *SE* = 83.75%, *F1* = 88.45%) as compared to other state-of-the-art approaches. This experimental result validates the effectiveness of the proposed model. Moreover, we observed that *gaus8* leads to higher classification performance in this study compared to other wavelet functions. However, the model is not lightweight because the training parameter is as high as about 34 million, which leads to high training time. In the future, we will research reducing the training parameter to make the proposed parallel cross convolutional RNN classifier lightweight and will investigate its performance on other physiological signal analysis. 

## Figures and Tables

**Table 1:** Configuration of the proposed network architecture.

Top Branch		Bottom Branch	
Layer	Output Volume	Layer	Output Volume
Input_1	(200, 1)	Input_2	(100, 100, 1)
Lstm_unit_1	(200, 16)	Conv_block_1	(50, 50, 16)
Reshape_1	(4, 50, 16)	-	-
Concatenate_1		(54, 50, 16)	
Reshape_2	(2700, 16)	-	-
Lstm_unit_2	(2700, 32)	Conv_block_2	(27, 25, 32)
Reshape_3	(108, 25, 32)	-	-
Concatenate_2		(135, 25, 32)	
Reshape_4	(3375, 32)	-	-
Lstm_unit_3	(3375, 64)	Conv_block_3	(68, 13, 64)
Flatten_1	(216000)	Flatten_2	(56576)
Concatenate_3		(272576)	
Dropout		(272576)	
Dense_1		(128)	
Dense_2		(4)	

**Table 2:** ECG class mapping using the AAMI standard.

AAMI Classes	Class Symbol	Heartbeat Types	Training Samples	Test Samples
Normal Beat	NB	Normal beat (NOR)	72,000	18,000
		Right bundle branch block beat (RBBB)		
		Left bundle branch block beat (LBBB)		
		Atrial escape beat (AE)		
		Nodal (junctional) escape beat (NE)		
Supraventricular Ectopic Beat	SVEB	Atrial premature beat (AP)	2160	540
		Premature or ectopic supraventricular beat (SP)		
		Nodal premature beat (NP)		
		Aberrated atrial premature beat (AAP)		
Ventricular Ectopic Beat	VEB	Ventricular escape beat (VE)	5600	1400
		Premature ventricular contraction (PVC)		
Fusion Beat	FB	Fusion of ventricular and normal beat (FVN)	641	160

**Table 3:** Hyper-parameter of the model training.

Hyper-Parameter	Values
Training data shape	(80401, 100, 100, 1) and (80401, 200, 1)
Test data shape	(20100, 100, 100, 1) and (20100, 200, 1)
Batch size	512
Learning rate	0.0005, 0.0001, 0.00005
Epoch	60
Optimizer	*Adam*
Loss function	Categorical-cross entropy

**Table 4:** Classification performance of the proposed model by varying learning rate (*lr*).

Classes	Metrics	*lr* = 0.0005	*lr* = 0.0001	*lr* = 0.00005
NB	ACC	99.25	99.48	99.37
	PPV	99.41	99.62	99.35
	SE	99.75	99.79	99.94
	F1	99.58	99.71	99.64
SVEB	ACC	99.52	99.67	99.63
	PPV	95.49	93.56	98.34
	SE	86.29	94.07	87.77
	F1	90.67	93.81	92.75
VEB	ACC	99.79	99.84	99.80
	PPV	99.13	99.49	99.49
	SE	97.79	98.28	97.71
	F1	98.45	98.89	98.59
FB	ACC	99.74	99.83	99.76
	PPV	81.29	93.71	91.17
	SE	86.88	83.75	77.50
	F1	83.99	88.45	83.78
Average	ACC	**99.58**	**99.71**	**99.64**
	PPV	**93.83**	**96.60**	**97.09**
	SE	**92.68**	**93.97**	**90.73**
	F1	**93.17**	**95.22**	**93.69**

**Table 5:** Ten-fold cross-validation score of our proposed model.

K-Folds	NB			SVEB			VEB			FB		
	PPV	SE	F1	PPV	SE	F1	PPV	SE	F1	PPV	SE	F1
**Fold = 1**	99.41	99.94	99.68	96.80	89.63	93.08	99.27	97.00	98.12	97.10	82.72	89.33
**Fold = 2**	99.51	99.80	99.66	97.54	88.15	92.61	98.14	98.00	98.07	85.19	86.25	85.71
**Fold = 3**	99.67	99.88	99.77	96.98	95.19	96.07	99.42	97.57	98.49	88.61	87.50	88.05
**Fold = 4**	99.33	99.92	99.62	98.73	86.67	92.31	98.83	96.43	97.61	78.95	75.00	76.92
**Fold = 5**	99.22	99.88	99.55	96.76	88.52	92.46	98.67	95.57	97.10	89.23	72.50	80.00
**Fold = 6**	99.40	99.81	99.61	99.40	99.81	99.61	98.70	97.57	98.13	88.46	85.19	86.79
**Fold = 7**	99.57	99.82	99.69	94.25	91.11	92.66	98.86	99.00	98.93	98.46	80.00	88.28
**Fold = 8**	99.60	99.78	99.69	94.74	93.68	94.21	97.58	98.00	97.79	95.31	76.25	84.72
**Fold = 9**	99.31	99.80	99.56	92.02	89.30	90.64	99.12	96.71	97.90	91.80	70.00	79.43
**Fold = 10**	99.57	99.78	99.67	93.04	94.07	93.55	98.56	97.86	98.21	93.65	73.75	82.52
**Average**	**99.46**	**99.84**	**99.65**	**96.03**	**91.61**	**93.72**	**98.72**	**97.37**	**98.04**	**90.68**	**78.92**	**84.18**

**Table 6:** Comparison of the classification performance of existing studies and our studies (*lr* = 0.0001) in terms of all classes.

Classes	Metrics	Other Studies						Ours
		Liu et al. [14]	Zhang et al. [11]	Ye et al. [12]	Wang et al. [26]	Jiang et al. [28]	Y. Zhang et al. [29]	
NB	PPV	96.66%	98.98%	97.55%	98.17%	98.39%	99.36%	**99.62%**
	SE	94.06%	88.94%	88.61%	99.42%	97.64%	99.62%	**99.79%**
	F1	95.34%	93.69%	92.87%	98.79%	98.01%	99.48%	**99.71%**
SVEB	PPV	39.87%	35.98%	52.34%	89.54%	63.70%	97.48%	**93.56%**
	SE	33.12%	79.06%	61.02%	74.56%	64.40%	98.44%	**94.07%**
	F1	36.18%	49.46%	56.34%	81.37%	64.04%	97.96%	**93.81%**
VEB	PPV	76.51%	92.75%	61.45%	93.25%	90.00%	92.66%	**99.49%**
	SE	90.20%	85.48%	81.82%	95.65%	91.00%	87.27%	**98.28%**
	F1	82.79%	88.96%	70.19%	94.43%	90.50%	89.88%	**98.89%**
FB	PPV	12.99%	13.73%	2.50%	2.04%	43.99%	91.04%	**93.71%**
	SE	40.72%	93.81%	19.69%	0.26%	76.70%	76.25%	**83.75%**
	F1	19.70%	23.96%	4.43%	0.46%	55.91%	82.99%	**88.45%**
Average	PPV	56.51%	60.36%	53.46%	70.75%	74.02%	95.14%	**96.60%**
	SE	64.53%	86.82%	62.79%	67.47%	82.44%	90.40%	**93.97%**
	F1	58.50%	64.02%	55.96%	68.76%	77.12%	92.58%	**95.22%**

**Table 7:** Comparison of the classification performance of the four wavelet functions in terms of all classes (a) NB class, (b) SVEB class, (c)VEB class, and (d) FB class.

Classes	Wavelet Functions	ACC	PPV	SE	F1
NB	*gaus8*	99.48	99.62	99.79	99.70
	*gaus4*	99.38	99.38	99.93	99.66
	*mexh*	99.50	99.53	99.91	99.72
	*morl*	99.52	99.63	99.84	99.73
SVEB	*gaus8*	99.67	93.55	94.07	93.81
	*gaus4*	99.63	98.74	87.40	92.73
	*mexh*	99.72	98.01	91.67	94.74
	*morl*	99.71	96.35	92.96	94.62
VEB	*gaus8*	99.84	99.49	98.28	98.88
	*gaus4*	99.82	98.92	98.50	98.71
	*mexh*	99.81	99.78	98.50	98.64
	*morl*	99.82	98.50	98.92	98.71
FB	*gaus8*	99.82	93.70	83.75	88.44
	*gaus4*	99.79	95.41	78.13	85.91
	*mexh*	99.81	95.55	80.62	87.48
	*morl*	99.77	94.53	75.62	84.02

## Data Availability

All the data used in this study are obtained from public datasets. Readers should be able to obtain these data by requesting the dataset sources described in this study.
